# Exosomal Circ-MEMO1 Promotes the Progression and Aerobic Glycolysis of Non-small Cell Lung Cancer Through Targeting MiR-101-3p/KRAS Axis

**DOI:** 10.3389/fgene.2020.00962

**Published:** 2020-08-28

**Authors:** Chengzhi Ding, Gaoyuan Xi, Guolei Wang, Dong Cui, Binbin Zhang, Hongtao Wang, Gongqian Jiang, Jingchao Song, Guanghui Xu, Jiao Wang

**Affiliations:** ^1^Department of Thoracic Surgery, Henan Provincial Chest Hospital, Zhengzhou, China; ^2^Department of Anesthesiology, Henan Provincial Chest Hospital, Zhengzhou, China; ^3^Department of Endocrinology, The First Affiliated Hospital of Zhengzhou University, Zhengzhou, China

**Keywords:** NSCLC, circ-MEMO1, miR-101-3p, KRAS, glycolysis, exosome

## Abstract

Circular RNA mediator of cell motility 1 (circ-MEMO1) was identified as an oncogene in non-small cell lung cancer (NSCLC). Nevertheless, the working mechanism behind circ-MEMO1-mediated progression of NSCLC is barely known. Quantitative real-time polymerase chain reaction (qRT-PCR) was applied to detect the expression of circ-MEMO1, microRNA-101-3p (miR-101-3p), and KRAS proto-oncogene, GTPase (KRAS). Cell proliferation and aerobic glycolysis were detected by 3-(4,5-Dimethylthiazol-2-yl)-2,5-diphenyltetrazolium bromide (MTT) assay and glycolysis detection kits. Flow cytometry was used to evaluate cell cycle progression and apoptosis of NSCLC cells. Western blot assay was used to measure the protein expression of hexokinase 2 (HK2), lactate dehydrogenase A (LDHA), KRAS, CD9, CD81, tumor susceptibility 101 (TSG101), and Golgi matrix protein 130 kDa (GM130). The target relationship between miR-101-3p and circ-MEMO1 or KRAS was predicted by StarBase software and confirmed by dual-luciferase reporter assay, RNA immunoprecipitation (RIP) assay, and RNA-pull down assay. *In vivo* tumor growth assay was conducted to assess the effect of circ-MEMO1 *in vivo*. Exosomes were isolated using the ExoQuick precipitation kit. Circ-MEMO1 was up-regulated in NSCLC, and high expression of circ-MEMO1 predicted poor prognosis in NSCLC patients. Circ-MEMO1 accelerated the proliferation, cell cycle progression, and glycolytic metabolism and inhibited the apoptosis of NSCLC cells. Circ-MEMO1 negatively regulated the expression of miR-101-3p through direct interaction, and si-circ-MEMO1-induced biological effects were attenuated by the introduction of anti-miR-101-3p. MiR-101-3p directly interacted with the 3′ untranslated region (3′ UTR) of KRAS messenger RNA (mRNA), and KRAS level was regulated by circ-MEMO1/miR-101-3p axis. Circ-MEMO1 silencing suppressed the NSCLC tumor growth *in vivo*. ROC curve analysis revealed that high expression of serum exosomal circ-MEMO1 (exo-circ-MEMO1) might be a valuable diagnostic marker for NSCLC. Circ-MEMO1 facilitated the progression and glycolysis of NSCLC through regulating miR-101-3p/KRAS axis.

## Introduction

The activation of pro-tumor genes and the inactivation of anti-tumor genes both contribute to the initiation and progression of cancers. KRAS proto-oncogene GTPase (KRAS) is a member of pro-tumor genes that always abnormally activated in diverse cancers (Chen et al., 2014). Several types of changes in KRAS gene result in the aberrant activation of KRAS, including KRAS gene mutation (codon 12 or 13), overexpression, and gene fusion (Hoa et al., 2002; Riely et al., 2009). Here we provided a novel signal axis in which KRAS was activated by its upstream genes and participated in the progression of non-small cell lung cancer (NSCLC).

Exosomes are small extracellular vesicles (30–150 nm) that are released by many types of cells. Exosomes mediate intercellular communication through the transfer of proteins, circular RNAs (circRNAs), microRNAs (miRNAs), and messenger RNAs (mRNAs) (Ostenfeld et al., 2014; Takahashi et al., 2014). Exosomal circ_0044516 accelerated the progression of prostate cancer by targeting miR-29a-3p (Li et al., 2020). Liu et al. (2020) revealed that circ-MMP2 could be transferred between different hepatocellular carcinoma (HCC) cell lines, and circ-MMP2 accelerated HCC cells motility via miR-136-5p/MMP2 axis. Here, we investigated the role of circ-MEMO1 and assessed the clinical diagnostic significance of serum exosomal circ-MEMO1 in NSCLC.

MiRNAs regulate gene expression through directly binding to mRNAs via their complementary sequences, thus causing the degradation or the translational suppression of mRNAs (Garzon et al., 2007). MiR-101-3p acted as a tumor suppressor in various cancers, including NSCLC. MiR-101-3p was found to be a target of SPRY4-IT1 to suppress the growth and motility of bladder cancer cells through down-regulating EZH2 (Liu et al., 2017). Cui et al. (2017) claimed that SNHG1 contributed to NSCLC development through targeting miR-101-3p. However, the interaction and regulatory relationship between miR-101-3p and circ-MEMO1 or KRAS have not been identified.

According to the clinical data, high levels of circ-MEMO1 might be a biomarker of poor prognosis in NSCLC patients. Through using bioinformatic software, the downstream genes of circ-MEMO1 were illustrated to disclose the network behind circ-MEMO1-mediated influence in NSCLC development.

## Materials and Methods

### Clinical Samples

NSCLC tissue samples (*n* = 52) and matching adjacent normal tissues (*n* = 52) were gathered from NSCLC patients at Henan Provincial Chest Hospital. Serum samples from NSCLC patients (*n* = 30) and healthy volunteers (*n* = 25) were also collected at Henan Provincial Chest Hospital. Written informed consent was provided from every subject. The protocol in this clinical experiment was authorized by the Clinical Research Ethics Committee of the Henan Provincial Chest Hospital and was carried out according to the guidelines of Declaration of Helsinki.

### Cell Lines

NSCLC cell lines (H1650, PC9, H1299, and A549) and normal human bronchial epithelial cell line (HBE) were obtained from BeNa Culture Collection (Beijing, China). These cell lines were both cultured with Roswell Park Memorial Institute-1640 (RPMI-1640) medium (Gibco, Carlsbad, CA, United States) supplemented with heat-inactivated 10% fetal bovine serum (FBS) and 10% penicillin (100 U/mL)/streptomycin (100 μg/mL) in a 37°C and 5% CO_2_ incubator.

### Quantitative Real-Time Polymerase Chain Reaction (qRT-PCR)

For exosomal RNA and cellular RNA extraction, miRNeasy Serum/Plasma kit (QIAGEN, Waltham, MA, United States) and Trizol solution (Invitrogen, Carlsbad, CA, United States) were used. After synthesizing template DNA, amplification reaction was conducted with specific primers and iQSYBR Green SuperMix (Bio-Rad, Hercules, CA, United States). U6 served as the house-keeping gene for miR-101-3p, and glyceraldehyde-3-phosphate dehydrogenase (GAPDH) acted as the endogenous reference for circ-MEMO1 and KRAS. The primers were listed in Table 1. The quantification was carried out with the 2^–ΔΔCt^ method.

**TABLE 1:** Primers used in qRT-PCR.

Gene name	Primer sequence
circ-MEMO1	CGTGACCCAGAAGTGCGTTCACA (forward) TGGGGGTGTATCAGTCTTTGGTT (reverse)
miR-101-3p	GCCGCCACCATGGTGAGCAAGG (forward) AATTGAAAAAAGTGATTTAATTT (reverse)
KRAS	TCTCCTTCTCAGGATTCCTACAG (forward) ACAAAGAAAGCCCTCCCCAGT (reverse)
U6	TGCGGGTGCTCGCTTCGGC (forward) CCAGTGCAGGGTCCGAGGT (reverse)
GAPDH	TGACCACAGTCCATGCCATC (forward) TTACTCCTTGGAGGCCATGT (reverse)

### RNase R and Actinomycin D Treatment

To suppress the transcription, 2 mg/mL actinomycin D was added to the culture medium. For RNase R treatment assay, RNA was digested using 3 U/μg RNase R or not for 30 min at room temperature. The abundance of circ-MEMO1 and GAPDH was examined by qRT-PCR experiment.

### Subcellular Fractionation

NSCLC cells were collected using ice-cold phosphate buffered saline (PBS) buffer, and total RNA sample was isolated with TRIzol solution (Invitrogen). Cytoplasmic and nuclear RNAs were distinguished using the PARIS™ Kit (Thermo Fisher Scientific, Waltham, MA, United States).

### Cell Transfection

Circ-MEMO1 specific small interfering RNA and short hairpin RNA (si-circ-MEMO1 and sh-circ-MEMO1) and their negative control (si-NC and sh-NC), miR-101-3p mimics (miR-101-3p), miR-101-3p inhibitor (anti-miR-101-3p), and their corresponding controls (miR-NC and anti-NC) were obtained from Genepharma (Shanghai, China).

### 3-(4,5-Dimethylthiazol-2-yl)-2,5-Diphenyltetrazolium Bromide (MTT) Assay

Briefly, after transfection for 24, 48, and 72 h, NSCLC cells were mixed with MTT reagent for 4 h at room temperature. To dissolve the reaction products after discarding the supernatant, 200 μL dimethylsulfoxide (DMSO) was used. The absorbance value was measured at 490 nm wave length.

### Flow Cytometry

For cell cycle analysis, NSCLC cells were fixed with 70% cold ethanol solution at −20°C. Subsequently, these NSCLC cells were dyed using propidium iodide (PI; Solarbio, Beijing, China) for 20 min at room temperature. The percentages of NSCLC cells in G0/G1 phase, S phase, or G2/M phase were analyzed by the flow cytometer.

For cell apoptosis analysis, after transfection for 72 h, NSCLC cells were collected. Fluorescein isothiocyanate (FITC) combined Annexin V and PI were used to stain NSCLC cells in a dark room for 15 min at room temperature. The apoptotic NSCLC cells in the early stage and late stage were identified using the flow cytometer.

### Glycolysis Analysis

After transfection for 48 h, the culture medium was collected. The glucose uptake and lactate production were detected using Glucose Assay Kit (Rsbio, Shanghai, China) and Lactate Assay Kit (Rsbio), respectively.

### Western Blot Assay

After transfection for 48 h, NSCLC cells were harvested. The cell lysates were obtained using the Radioimmunoprecipitation assay (RIPA) lysis buffer (Beyotime, Shanghai, China). Cell lysates (30 μg) were subjected to 12% separating gel and then blotted to the polyvinylidene fluoride (PVDF) membrane (Bio-Rad). After blocking, the PVDF membrane was mixed with the primary antibodies, including anti-hexokinase 2 (anti-HK2, ab209847, Abcam, Cambridge, MA, United States), anti-lactate dehydrogenase A (anti-LDHA, ab101562, Abcam), anti-KRAS (ab180772, Abcam), anti-CD9 (ab92726, Abcam), anti-CD81 (ab79559, Abcam), anti-tumor susceptibility 101 (anti-TSG101, ab125011, Abcam), anti-Golgi matrix protein 130 kDa (anti-GM130, ab32337, Abcam), and anti-β-actin (anti-20272, Abcam). After washing three times using PBS-Tween 20 (PBST), horseradish peroxidase(HRP)-combined secondary antibody (ab205718, Abcam) was utilized to incubate with the membrane for 2 h at room temperature. After washing with PBST, protein signals were visualized using the enhanced chemiluminescent (ECL) system (Beyotime).

### Dual-Luciferase Reporter Assay

StarBase database was used to predict the interactions of circ-MEMO1-miRNAs and miR-101-3p-mRNAs. Dual-luciferase reporter assay was used to test the target relationship between miR-101-3p and circ-MEMO1 or KRAS. The partial sequence in circ-MEMO1 or the 3′ untranslated region (3′ UTR) of KRAS messenger RNA (mRNA), containing the binding sites with miR-101-3p, was directly amplified and inserted into pGL3 luciferase reporter vector (Promega, Madison, WI, United States), termed as circ-MEMO1-wt or KRAS 3′ UTR wt. Meanwhile, the corresponding mutant sequence in circ-MEMO1 or KRAS was also amplified and inserted into pGL3 luciferase reporter vector (Promega) to generate circ-MEMO1-mut or KRAS 3′ UTR mut using Site-directed gene mutagenesis kit (Takara, Dalian, China). After co-transfecting these reporter constructed plasmids (50 ng) and miR-NC or miR-101-3p (20 nM) into NSCLC cells for 48 h, the luciferase activity was detected with the dual-luciferase reporter assay system (Promega) using the luminometer (Plate Chameleon V, Hidex, Finland). Firefly luciferase activity was normalized to Renilla luciferase intensity. The experiment was repeated three times.

### RNA Immunoprecipitation (RIP) Assay

A Magna RIP™ RNA-Binding Protein Immunoprecipitation kit (Millipore, Billerica, MA, United States) was used to conduct RIP assay. Cell lysates were divided into two equal parts, and these two parts were incubated with Argonaute-2 (Ago2) antibody and Immunoglobulin G (IgG) antibody, respectively. Sepharose beads (Bio-Rad) were then added to incubate with the mixture. The expression of circ-MEMO1 and miR-101-3p was detected by qRT-PCR.

### RNA-Pull Down Assay

RNA-pull down assay was performed for the confirmation of the binding relationship between circ-MEMO1 and miR-101-3p. MiR-101-3p was biotinylated to generate bio-miR-101-3p, and bio-NC was used as the control. A total of 2 μg cell lysate was mixed with bio-miR-101-3p (100 pmol) or bio-NC (100 pmol). qRT-PCR was implemented for expression determination of circ-MEMO1.

### *In vivo* Tumor Growth Assay

Animal manipulates were authorized by the Animal Research Ethics Committee of Henan Provincial Chest Hospital. All animals received humane care according to the National Institutes of Health (United States) guidelines. Five-week-old BALB/c nude mice were arbitrarily divided into two groups (*n* = 7). Into the nude mice, 2 × 10^6^ A549 cells in 100 μL PBS transfected with sh-NC or sh-circ-MEMO1 were subcutaneously inoculated. These nude mice were routinely maintained for 28 days. After injection for 7 days, the volume of tumors in mice was measured using a vernier caliper with the formula length × width^2^ × 0.5, and the volume of tumors was continuously measured every 3 days to generate the tumor growth curve. After injection for 28 days, the tumors were resected and the weight was measured using the analytical balance.

### Exosome Isolation

Exosomes were extracted from the serum samples of NSCLC patients and healthy volunteers using the ExoQuick precipitation kit (System Biosciences, Mountain View, CA, United States). The exosomes-containing pellets were suspended using PBS and used for characterization via electron microscope and Western blot assay and RNA detection via qRT-PCR.

### Statistical Analysis

The results were displayed as mean ± standard deviation (SD). Two-tailed Student’s *t*-test and one-way analysis of variance (ANOVA) followed by Tukey’s test were used to compare the differences in two groups and multiple groups, respectively. Kaplan–Meier survival curve was analyzed with log-rank test. The receiver operating characteristic (ROC) curve analysis was conducted to measure the area under the curve (AUC) to evaluate the diagnostic value of serum exosomal circ-MEMO1 (exo-circ-MEMO1). A *P*-value less than 0.05 was considered statistically significant.

## Results

### High Expression of Circ-MEMO1 Is Associated With Poor Prognosis of NSCLC Patients

**FIGURE 1:**
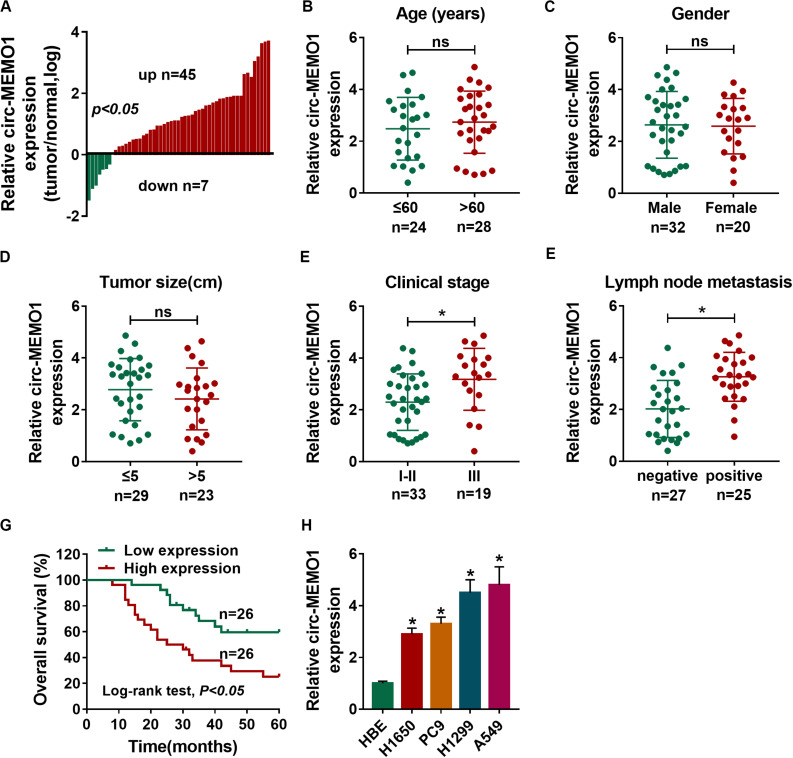
High expression of circ-MEMO1 is associated with poor prognosis of NSCLC patients. **(A)** The relative expression of circ-MEMO1 in NSCLC tumor tissues (*n* = 52) and adjacent normal tissues (*n* = 52) was detected by qRT-PCR, and the ratio of circ-MEMO1 expression in tumor tissues and normal tissues was analyzed. **(B–F)** NSCLC patients were divided into two groups according to the age, gender, tumor size, clinical stage, or lymph node metastasis, and the expression of circ-MEMO1 in tumor tissues of each group was analyzed. **(G)** NSCLC patients were split into circ-MEMO1 high expression group (*n* = 26) and low expression group (*n* = 26) according to the median of circ-MEMO1 level, and the overall survival curve was generated by Kaplan–Meier plot and analyzed by log-rank test. **(H)** Circ-MEMO1 level was examined in human bronchial epithelial cell line (HBE) and NSCLC cell lines (H1650, PC9, H1299, and A549) by qRT-PCR. *P < 0.05.

A total of 52 NSCLC patients were enrolled in our study. We analyzed the expression of circ-MEMO1 in NSCLC tissues and adjacent normal tissues to explore if circ-MEMO1 was dysregulated in NSCLC. The ratio of circ-MEMO1 expression in tumor tissues and normal tissues in each participant was shown in Figure 1A. There were 45 NSCLC patients with higher expression of circ-MEMO1 in tumor tissues than that in normal tissues (Figure 1A). In addition, circ-MEMO1 was down-regulated in tumor tissues in a total of seven NSCLC patients compared with adjacent normal tissues (Figure 1A). These findings suggested that circ-MEMO1 was up-regulated in tumor tissues in most of the NSCLC patients and hinted that circ-MEMO1 might exert a pivotal role in NSCLC. To explore the correlation between circ-MEMO1 expression and the pathological features (age, gender, tumor size, clinical stage, and lymph node metastasis) of NSCLC patients, we divided these 52 NSCLC patients into two groups according to the above variables. As shown in Figures 1B–F, high expression of circ-MEMO1 was correlated with the advanced clinical stage and positive lymph node metastasis but not that of age, gender, and tumor size. NSCLC patients were divided into high circ-MEMO1 expression group and circ-MEMO1 low expression group according to the median value of circ-MEMO1 expression. Through tracking the survival time of these NSCLC patients, we generated survival curve in the two groups. Patients with higher expression of circ-MEMO1 possessed lower survival time (Figure 1G). Furthermore, the expression of circ-MEMO1 was also examined in HBE cell line and a panel of four NSCLC cell lines (H1650, PC9, H1299, and A549) by qRT-PCR. The level of circ-MEMO1 was higher in NSCLC cell lines compared with HBE cell line (Figure 1H). Taken together, high expression of circ-MEMO1 predicted poor prognosis of NSCLC patients.

### Circ-MEMO1 Stably Distributes in Cytoplasmic Fraction of NSCLC Cells

**FIGURE 2:**
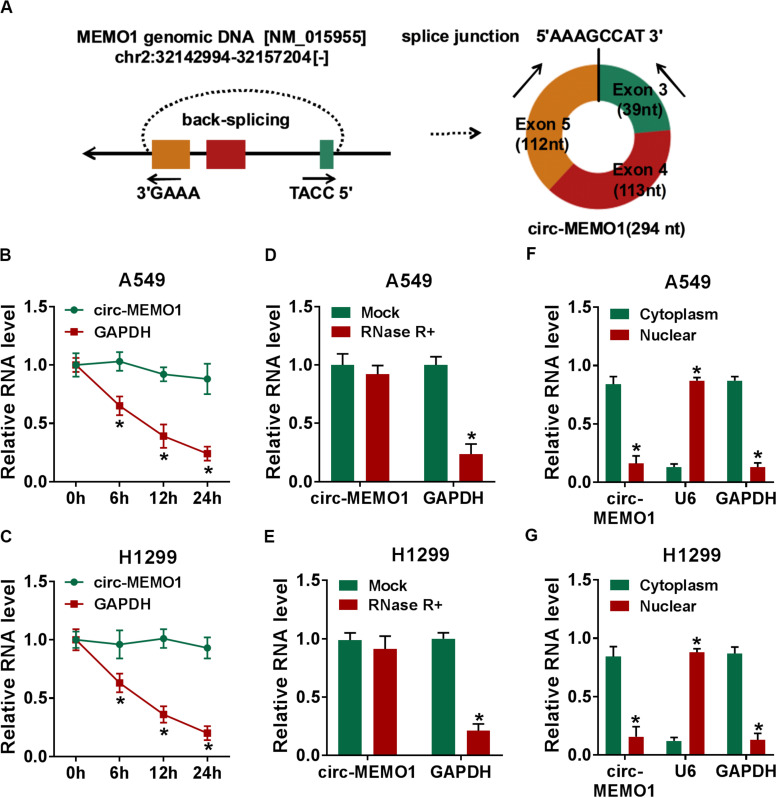
Circ-MEMO1 stably distributes in cytoplasmic fraction of NSCLC cells. **(A)** The schematic diagram revealed the formation of circ-MEMO1 from MEMO1 gene. Back-splicing occurs in the end of exon 3 and exon 5 to allow these three exons to form the closed loop structure. **(B,C)** Actinomycin D was used to inhibit transcription to test the stability of circ-MEMO1, and GAPDH served as the internal reference. qRT-PCR was used to detect the expression of circ-MEMO1 and GAPDH. **(D,E)** RNase was used to test the circular structure of circ-MEMO1, and qRT-PCR was conducted to detect the expression of circ-MEMO1 and GAPDH in A549 and H1299 cells with or without RNase treatment. **(F,G)** qRT-PCR was performed to measure the expression of circ-MEMO1 in cytoplasmic fraction and nuclear fraction of NSCLC cells. *P < 0.05.

Circ-MEMO1 generated from the exon 3, exon 4, and exon 5 of MEMO1 gene, and the end of exon 3 and exon 5 was back-spliced to form the circular structure (Figure 2A). Actinomycin D (transcription inhibitor) and RNase R were used to test the stability of circ-MEMO1. As shown in Figures 2B,C, after actinomycin D treatment, circ-MEMO1 was more stable in NSCLC cells compared with GAPDH. RNase R treatment significantly decreased the expression of GAPDH, while it almost had no effect on the expression of circ-MEMO1 compared with the untreated group (Figures 2D,E). To uncover the functions of circ-MEMO1 in NSCLC cells, we initially examined its distribution in nuclear fraction and cytoplasmic fraction of NSCLC cells. U6 served as the nuclear marker while GAPDH acted as the cytoplasmic marker. As shown in Figures 2F,G, circ-MEMO1 was mainly distributed in the cytoplasmic fraction of NSCLC cells. Collectively, circ-MEMO1 possessed the stable circular structure and it mainly located in the cytoplasmic fraction of NSCLC cells.

### Circ-MEMO1 Silencing Restrains Cell Proliferation and Cell Cycle Progression and Triggers Cell Apoptosis in NSCLC Cells

**FIGURE 3:**
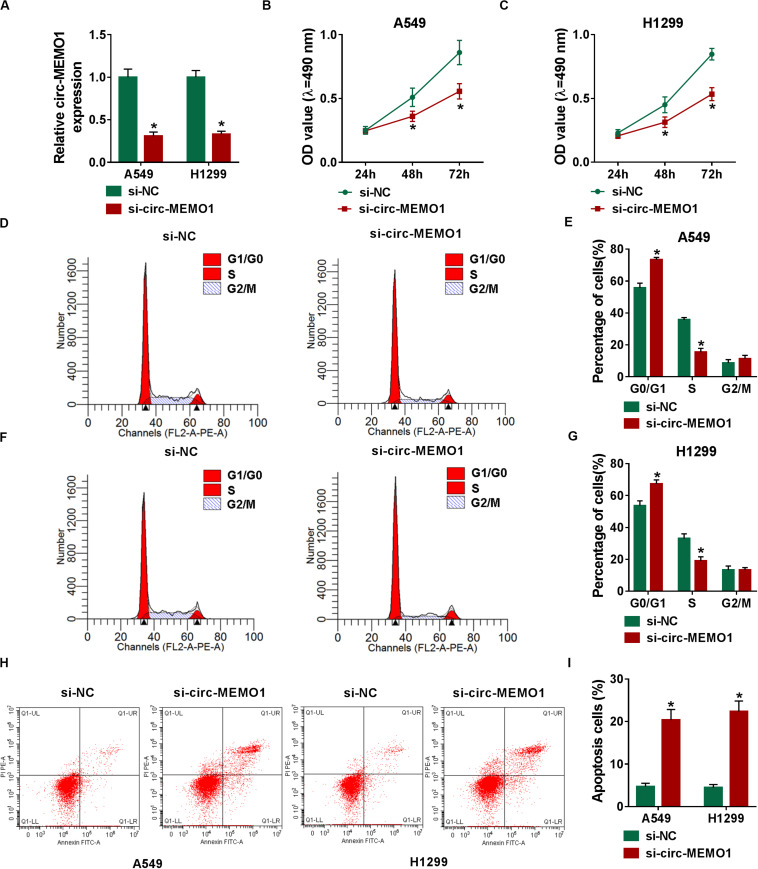
Circ-MEMO1 silencing restrains cell proliferation and cell cycle progression and triggers cell apoptosis in NSCLC cells. **(A–I)** A549 and H1299 cells were transfected with si-NC or si-circ-MEMO1. **(A)** Circ-MEMO1 level was detected in transfected NSCLC cells by qRT-PCR. **(B,C)** MTT assay was utilized to analyze the proliferation of NSCLC cells. **(D–G)** Flow cytometry was used to analyze the percentage of NSCLC cells in G0/G1, S, or G2/M phase with the silencing of circ-MEMO1 or not. **(H,I)** The percentage of apoptotic NSCLC cells in the early stage and late stage was analyzed by flow cytometry. *P < 0.05.

The specifically synthesized siRNA targeting circ-MEMO1 (si-circ-MEMO1) was used to conduct loss-of-function experiments. Si-circ-MEMO1 introduction notably decreased the level of circ-MEMO1 in A549 and H1299 cells (Figure 3A). MTT assay and flow cytometry were subsequently conducted to explore the functions of circ-MEMO1 on the proliferation, cell cycle, and apoptosis of NSCLC cells. The proliferation of NSCLC cells was suppressed with the interference of circ-MEMO1 (Figures 3B,C). The percentage of NSCLC cells in G0/G1 phase was notably increased while the percentage of cells in S phase was significantly decreased with the knockdown of circ-MEMO1 (Figures 3D–G), which suggested that circ-MEMO1 interference suppressed the cell cycle of NSCLC cells at G1/S transition. We counted the NSCLC cells in early stage and late stage of apoptosis in si-NC group and si-circ-MEMO1 group to test the role of circ-MEMO1 in the apoptosis of NSCLC cells. As shown in Figures 3H,I, si-circ-MEMO1 transfection promoted the apoptosis of NSCLC cells. These findings together demonstrated that circ-MEMO1 silencing restrained the proliferation and cell cycle progression and induced the apoptosis of NSCLC cells.

To further confirm the roles of circ-MEMO1 in the proliferation, cell cycle progression, and apoptosis of NSCLC cells, we conducted gain-of-function experiments using a H1650 cell line that had low expression of circ-MEMO1 compared with the other three NSCLC cell lines (PC9, H1299, and A549; shown in Figure 1H). The overexpression efficiency of circ-MEMO1 was high in H1650 cells (Supplementary Figure S1A). According to the results of MTT assay, circ-MEMO1 overexpression promoted the proliferation ability of H1650 cells (Supplementary Figure S1B). The percentage of circ-MEMO1-overexpressed H1650 cells in S phase was notably increased, while circ-MEMO1 overexpression reduced the percentage of H1650 cells in G0/G1 phase (Supplementary Figure S1C), suggested that circ-MEMO1 overexpression promoted cell cycle progression. The apoptosis rate was significantly reduced with the overexpression of circ-MEMO1 over that in the vector group (Supplementary Figure S1D). These findings manifested that circ-MEMO1 overexpression promoted the proliferation, cell cycle progression, and suppressed the apoptosis of NSCLC cells.

### Circ-MEMO1 Knockdown Hampers the Glycolysis of NSCLC Cells

**FIGURE 4:**
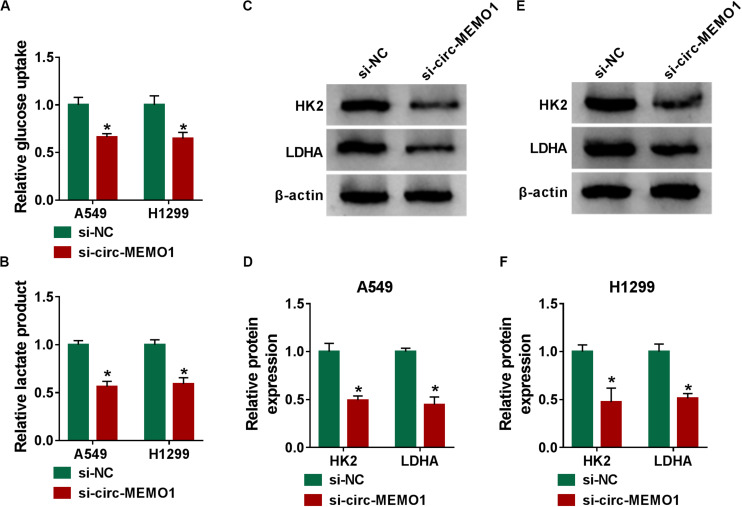
Circ-MEMO1 knockdown hampers the glycolysis of NSCLC cells. **(A–F)** A549 and H1299 cells were transfected with si-circ-MEMO1 or si-NC, respectively. **(A,B)** The glucose uptake and lactate production were measured by Glucose Assay Kit and Lactate Assay Kit. **(C–F)** The levels of glycolysis-related proteins (HK2 and LDHA) were examined in transfected NSCLC cells by Western blot assay, and the protein quantification was performed using Image J software. *P < 0.05.

Glycolysis is the major way by which cancer cells obtain energy, and the increase in the glucose uptake and lactate production is an important feature for activated glycolysis (Marchiq and Pouyssegur, 2016). We also tested the influence of circ-MEMO1 silencing on the glycolysis of NSCLC cells through measuring the consumption of glucose, the production of lactate, and the levels of glycolysis-related enzymes (HK2 and LDHA). Circ-MEMO1 knockdown down-regulated the glucose uptake and lactate production in NSCLC cells (Figures 4A,B). As shown in Figures 4C–F, circ-MEMO1 knockdown down-regulated the protein expression of HK2 and LDHA in NSCLC cells. As displayed in Supplementary Figures S1E,F, circ-MEMO1 overexpression promoted the glucose uptake and elevated the level of lactate in H1650 cells. Also, circ-MEMO1 overexpression up-regulated the expression of HK2 and LDHA in H1650 cells (Supplementary Figure S1G). Taken together, circ-MEMO1 accelerated the glycolysis of NSCLC cells.

### Circ-MEMO1 Directly Interacts With MiR-101-3p in NSCLC Cells

**FIGURE 5:**
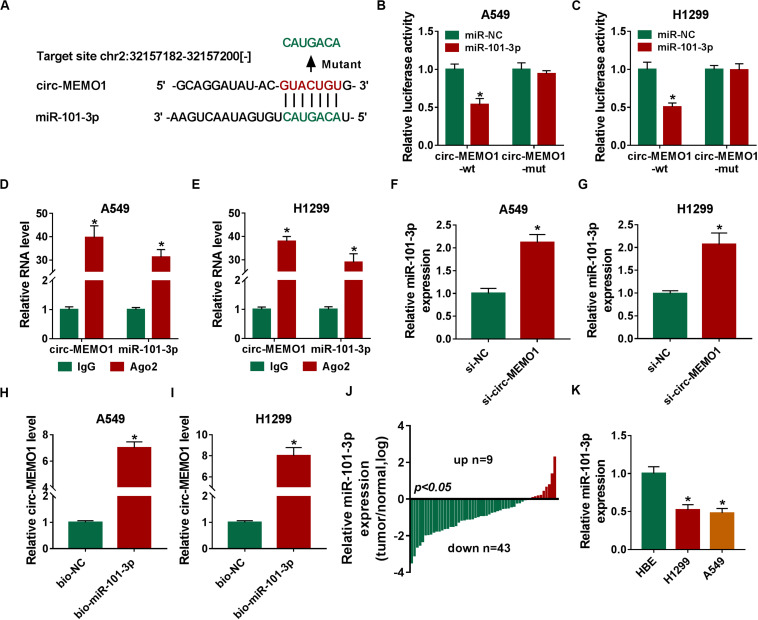
Circ-MEMO1 directly interacts with miR-101-3p in NSCLC cells. **(A)** The binding sequence between circ-MEMO1 and miR-101-3p was predicted by StarBase software, and the binding sites in circ-MEMO1 (GUACUGU) were mutated by CAUGACA to perform dual-luciferase reporter assay. **(B,C)** Dual-luciferase reporter assay was conducted to verify the direct target interaction between miR-101-3p and circ-MEMO1. NSCLC cells were transfected with the following four groups: miR-NC + circ-MEMO1-wt, miR-101-3p + circ-MEMO1-wt, miR-NC + circ-MEMO1-mut, and miR-101-3p + circ-MEMO1-mut. **(D,E)** RIP assay was utilized to confirm the spatial interaction between miR-101-3p and circ-MEMO1 in NSCLC cells, and IgG group was used as the control group. **(F,G)** The abundance of miR-101-3p was analyzed in NSCLC cells transfected with si-NC or si-circ-MEMO1 by qRT-PCR. **(H,I)** RNA-pull down assay was conducted to verify the interaction between miR-101-3p and circ-MEMO1 using biotinylated miR-101-3p (bio-miR-101-3p) or bio-NC in A549 and H1299 cells. **(J)** The ratio of miR-101-3p expression in tumor samples (*n* = 52) and corresponding normal samples (*n* = 52) was analyzed. **(K)** qRT-PCR was applied to detect the abundance of miR-101-3p in HBE cell line and NSCLC cell lines. *P < 0.05.

To uncover the potential mechanism by which circ-MEMO1 functioned, the possible miRNA targets of circ-MEMO1 were predicted using StarBase database. As shown in Figure 5A, miR-101-3p was predicted as a candidate target of cir-MEMO1, and the predicted binding sequence was also shown. To verify the interaction between miR-101-3p and circ-MEMO1, the fragment of circ-MEMO1, including the wild-type binding sites with miR-101-3p, was amplified and inserted into luciferase reporter vector to generate circ-MEMO1-wt. Circ-MEMO1-wt was co-transfected with miR-101-3p or miR-NC into NSCLC cells, and the luciferase activity was significantly decreased in miR-101-3p and circ-MEMO1-wt co-transfected group compared with miR-NC and circ-MEMO1-wt group (Figures 5B,C). The mutant type binding sequence in circ-MEMO1 was also inserted into luciferase reporter vector to generate circ-MEMO1-mut, and the luciferase activity remained almost unchanged in circ-MEMO1-mut group when co-transfected with miR-NC or miR-101-3p (Figures 5B,C), suggested that circ-MEMO1 directly targeted miR-101-3p in NSCLC cells. Ago2 is the core component of RNA-induced silencing complex (RISC), and miRNAs are known to bind to RISC. RIP assay was conducted to test the interaction between miR-101-3p and circ-MEMO1 using Ago2 antibody to pull down RISC, and IgG group was used as the control. As shown in Figures 5D,E, miR-101-3p interacted with circ-MEMO1 in NSCLC cells. The expression of miR-101-3p was notably increased with the interference of circ-MEMO1 in NSCLC cells (Figures 5F,G). The results of RNA-pull down assay revealed that circ-MEMO1 was pulled-down when using biotinylated miR-101-3p (bio-miR-101-3p) rather than bio-NC in A549 and H1299 cells (Figures 5H,I). As shown in Figure 5J, miR-101-3p was down-regulated in tumor tissues compared with normal tissues in most of the NSCLC patients. Furthermore, there was a notable decrease in the level of miR-101-3p in NSCLC cell lines compared with HBE cell line (Figure 5K). Overall, circ-MEMO1 down-regulated miR-101-3p in NSCLC cells through direct interaction.

### MiR-101-3p Knockdown Partly Alleviates si-circ-MEMO1-Mediated Effects in NSCLC Cells

**FIGURE 6:**
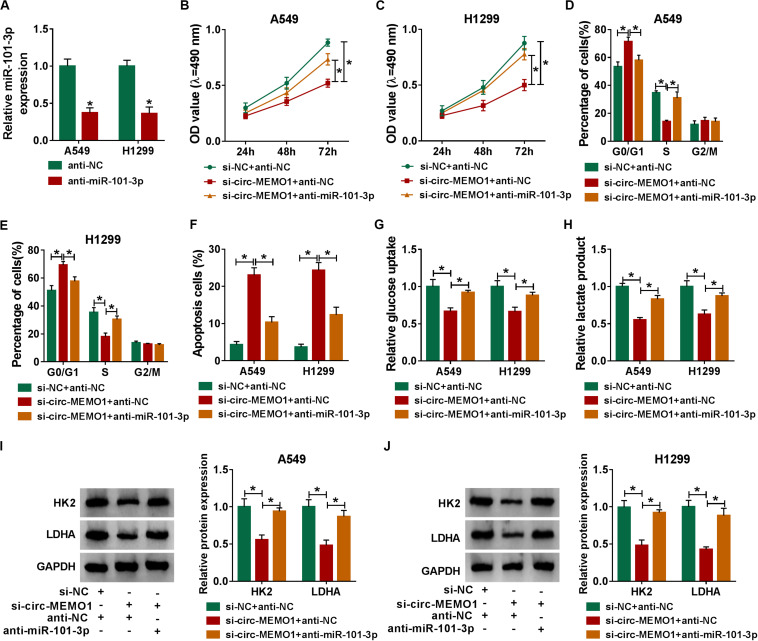
MiR-101-3p knockdown partly alleviates si-circ-MEMO1-mediated effects in NSCLC cells. **(A)** The knockdown efficiency of anti-miR-101-3p was assessed by qRT-PCR. **(B–J)** We transfected si-NC + anti-NC, si-circ-MEMO1 + anti-NC, or si-circ-MEMO1 + anti-miR-101-3p into NSCLC cells. **(B,C)** The proliferation ability of transfected NSCLC cells was evaluated by MTT assay. **(D,E)** The cell cycle of NSCLC cells was analyzed by flow cytometry. **(F)** The apoptosis rate of NSCLC cells was analyzed by flow cytometry. **(G,H)** The aerobic glycolysis of NSCLC cells was analyzed using Glucose Assay Kit and Lactate Assay Kit. **(I,J)** The expression of HK2 and LDHA in transfected NSCLC cells was detected by Western blot assay. *P < 0.05.

The knockdown efficiency of anti-miR-101-3p was high in NSCLC cells (Figure 6A). Rescue experiments were performed through transfecting si-NC + anti-NC, si-circ-MEMO1 + anti-NC, or si-circ-MEMO1 + anti-miR-101-3p into NSCLC cells. Circ-MEMO1 interference inhibited the proliferation (Figures 6B,C) and cell cycle (Figures 6D,E) while it promoted the apoptosis (Figure 6F) of NSCLC cells. Furthermore, the addition of anti-miR-101-3p recovered the malignant phenotypes of NSCLC cells (Figures 6B–F). Also, miR-101-3p knockdown counteracted the inhibitory effect of circ-MEMO1 silencing on the glycolysis of NSCLC cells (Figures 6G,H). The levels of HK2 and LDHA in NSCLC cells were notably decreased with the silencing of circ-MEMO1, and these influences were attenuated by the introduction of anti-miR-101-3p (Figures 6I,J). Overall, circ-MEMO1 exerted an oncogenic role in NSCLC cells through targeting miR-101-3p.

### MiR-101-3p Directly Interacts With the 3′’ UTR of KRAS in NSCLC Cells

**FIGURE 7:**
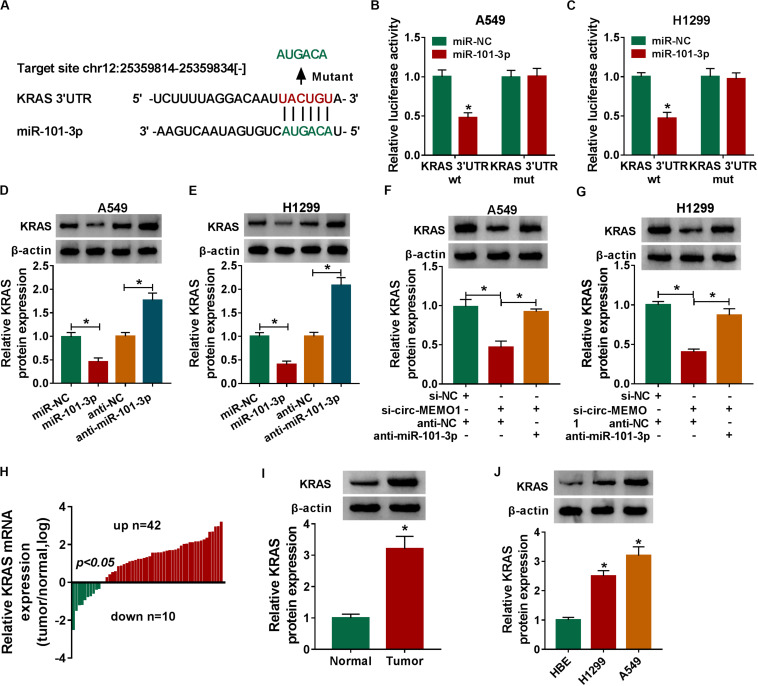
MiR-101-3p directly interacts with the 3′ UTR of KRAS in NSCLC cells. **(A)** The interactions of miR-101-3p-mRNAs were screened by StarBase software, and KRAS was a candidate target of miR-101-3p. The mutant binding sites with miR-101-3p in KRAS were also shown. **(B,C)** Dual-luciferase reporter assay was performed to test the direct interaction between miR-101-3p and KRAS in NSCLC cells. **(D,E)** The regulatory relationship between miR-101-3p and KRAS in NSCLC cells was analyzed through transfecting miR-101-3p, anti-miR-101-3p, and their negative controls into NSCLC cells, and then the expression of KRAS was measured via Western blot assay. **(F,G)** The expression of KRAS was detected in A549 and H1299 cells co-transfected with si-NC + anti-NC, si-circ-MEMO1 + anti-NC, or si-circ-MEMO1 + anti-miR-101-3p by Western blot assay. **(H)** The expression of KRAS mRNA in NSCLC tumor tissues and matching normal tissues was examined by qRT-PCR assay. **(I)** Western blot assay was used to detect the protein expression of KRAS in NSCLC tumor tissue and matching normal tissue. **(J)** The expression of KRAS in HBE, H1299, and A549 cell lines was detected by Western blot assay. *P < 0.05.

KRAS was predicted to be a direct target of miR-101-3p by StarBase software, and the binding sites between these two genes were shown in Figure 7A. MiR-101-3p transfection down-regulated the luciferase activity in KRAS 3′ UTR wt group compared with that in miR-NC and KRAS 3′ UTR wt group (Figures 7B,C), which suggested that miR-101-3p directly interacted with the 3′ UTR of KRAS mRNA in NSCLC cells. We transfected miR-101-3p and anti-miR-101-3p into NSCLC cells to explore the regulatory relationship between miR-101-3p and KRAS. As shown in Figures 7D,E, miR-101-3p overexpression decreased the level of KRAS protein, while miR-101-3p silencing up-regulated the level of KRAS in NSCLC cells. Furthermore, the relationship among circ-MEMO1, miR-101-3p, and KRAS was explored. As shown in Figures 7F,G, circ-MEMO1 silencing down-regulated the expression of KRAS, and the addition of anti-miR-101-3p recovered the expression of KRAS in NSCLC cells. KRAS mRNA level was significantly up-regulated in NSCLC tumor tissues compared with adjacent normal tissues in a total of 42 NSCLC patients (Figure 7H). The protein level of KRAS in tumor tissues (*n* = 3) was also higher than that in normal tissues (*n* = 3) (Figure 7I). High expression of KRAS protein was also found in H1299 and A549 cell lines compared with that in HBE cell line (Figure 7J). Collectively, KRAS mRNA was a direct target of miR-101-3p in NSCLC cells.

### Circ-MEMO1 Interference Restrains the NSCLC Tumor Growth *in vivo*

**FIGURE 8:**
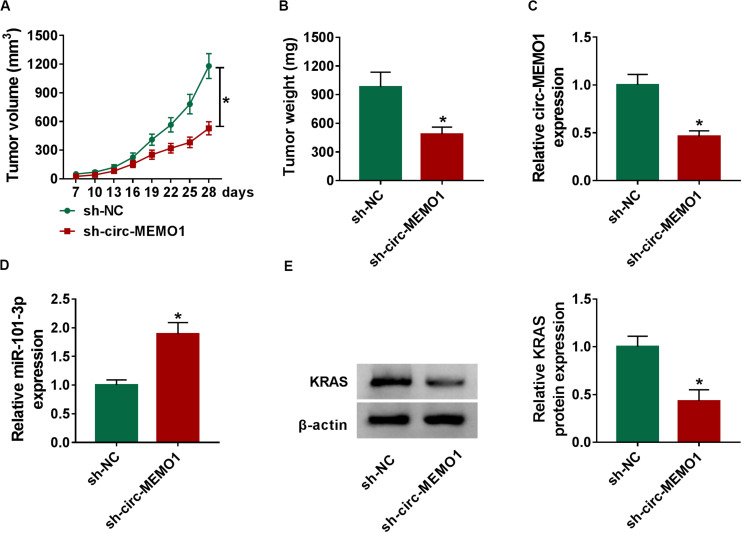
Circ-MEMO1 interference restrains the NSCLC tumor growth *in vivo*. BALB/c nude mice were divided into sh-NC group (*n* = 7) and sh-circ-MEMO1 group (*n* = 7). A549 cells stably expressing sh-NC or sh-circ-MEMO1 were subcutaneously injected into the right back of the nude mice in the corresponding group. **(A)** The tumor volume was measured after injection for 7 days every 3 days with the formula: length × width^2^ × 0.5. **(B)** Tumor weight in sh-NC group and sh-circ-MEMO1 group was measured with an analytical balance. **(C,D)** The expression of circ-MEMO1 and miR-101-3p in two groups was detected by qRT-PCR. **(E)** Western blot assay was used to detect the protein expression of KRAS in two groups. *P < 0.05.

To explore the function of circ-MEMO1 in the NSCLC tumor growth *in vivo*, we injected A549 cells stably expressing sh-NC or sh-circ-MEMO1 into the nude mice. Tumors in sh-circ-MEMO1 group were smaller than those in sh-NC group (Figures 8A,B). The expression of circ-MEMO1, miR-101-3p, and KRAS in tumor tissues was detected by Western blot assay and qRT-PCR. The abundance of circ-MEMO1 and the protein expression of KRAS were decreased in sh-circ-MEMO1 group compared with sh-NC group (Figures 8C,E). Furthermore, miR-101-3p level was higher in tumor tissues with the silencing of circ-MEMO1 compared with sh-NC group (Figure 8D). These findings suggested that circ-MEMO1 knockdown suppressed the growth of NSCLC tumors *in vivo*.

### Exosomal Circ-MEMO1 Is Higher in the Serum From NSCLC Patients Compared With Healthy Volunteers

**FIGURE 9:**
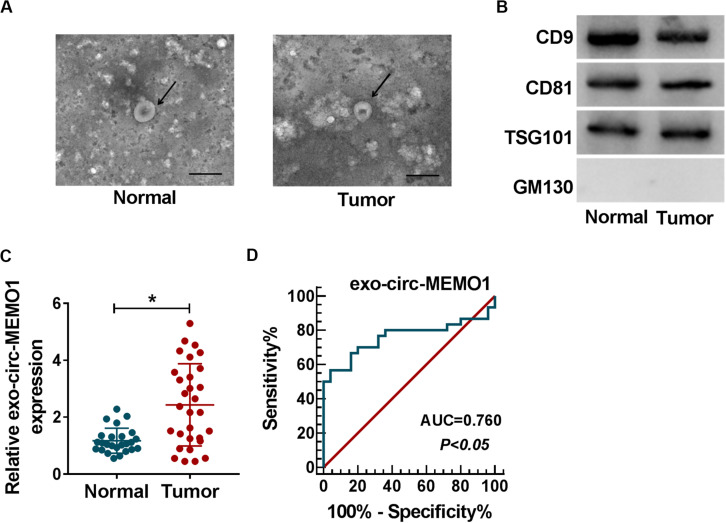
Exosomal circ-MEMO1 is higher in the serum from NSCLC patients compared with healthy volunteers. **(A)** The electron microscopy images showed the exosomes isolated from serum of NSCLC patient and healthy volunteer. Ruler length indicates 200 nm. **(B)** The expression of exosomal markers (CD9, CD81, and TSG101) and Golgi matrix protein (GM130) was detected by Western blot assay. **(C)** The expression of exosomal circ-MEMO1 in the serum from NSCLC patients (*n* = 30) and healthy volunteers (*n* = 25) was detected by qRT-PCR. **(D)** ROC curve was generated to evaluate the diagnostic value of exosomal circ-MEMO1 level in serum of NSCLC patients. *P < 0.05.

To explore if exosomes were involved in circ-MEMO1-mediated progression of NSCLC, we conducted further experiments. Exosomes were isolated from the serum of NSCLC patients (*n* = 30) and healthy volunteers (*n* = 25). The representative images of exosomes from NSCLC patients and healthy volunteers were shown in Figure 9A. To verify the characteristics of exosomes, we detected exosome markers and Golgi matrix protein (GM130) in isolated exosomes. The expression of CD9, CD81, and TSG101 was high in exosomes from the serum of NSCLC patients and healthy volunteers, while there was almost no expression of GM130 in isolated exosomes (Figure 9B). Circ-MEMO1 was higher in exosomes generated from the serum of NSCLC patients compared with healthy volunteers (Figure 9C). Furthermore, the receiver operating characteristic (ROC) curve was used to assess the diagnostic value of exo-circ-MEMO1 in serum. The area under curve (AUC) of the ROC curve reached about 0.76, with the diagnostic sensitivity and specificity of 56.67 and 96%, respectively (95% confidence interval [CI] = 0.6259–0.8941) (Figure 9D). Altogether, serum exo-circ-MEMO1 might be a promising biomarker for NSCLC treatment.

## Discussion

CircRNAs are novel circular non-coding RNAs, and they are closely related to the initiation and progression of multiple cancers (Meng et al., 2017; Patop and Kadener, 2018; Zhang et al., 2018). The present study focused on the biological role and the mechanism of circ-MEMO1 in NSCLC progression. In addition, the diagnostic value of exosomal circ-MEMO1 level in serum was also assessed.

Accumulating circRNAs were found to act as important regulators in human cancers and diseases with the development and application of sequencing technology (Dang et al., 2017; Qu et al., 2018). For instance, circ_0005075 accelerated the progression of colorectal cancer (Zhong et al., 2019). Circ_0000993 suppressed the motility of gastric cancer cells (Zhong et al., 2018). Circ-MEMO1 was found to be an oncogene in NSCLC through conducting microarray profiles (Jiang et al., 2018). Consistent with former article (Jiang et al., 2018), circ-MEMO1 was found to be up-regulated in NSCLC tissues through analyzing the clinical data, and high abundance of circ-MEMO1 was closely related to the dismal prognosis of NSCLC patients, suggesting that circ-MEMO1 might be a promising prognosis marker for NSCLC. Through conducting loss-of-function experiments and gain-of-function experiments, we found that circ-MEMO1 accelerated the proliferation, cell cycle progression, and glycolysis while suppressing the apoptosis of NSCLC cells.

CircRNAs mainly function in human cancers and diseases through targeting corresponding miRNAs to up-regulate the expression of mRNAs. For instance, circ-BPTF accelerated the development of bladder cancer through up-regulating RAB27A via targeting miR-31-5p (Bi et al., 2018). Circ_0071589 facilitated the progression of colorectal cancer through enhancing the level of EZH2 through targeting miR-600 (Yong et al., 2018). Through using StarBase software, we found that miR-101-3p was a candidate target miRNA of circ-MEMO1, and this direct interaction was verified by dual-luciferase reporter assay, RIP assay, and RNA-pull down assay. The biological role of miR-101-3p in diverse cancers has been reported. MiR-101-3p enhanced the oxaliplatin sensitivity of hepatocellular carcinoma cells (Sun et al., 2019). Meng et al. (2018) reported that SNHG6 accelerated glioma progression through targeting and down-regulating miR-101-3p. Consistent with these articles, we found that miR-101-3p played an anti-tumor role in NSCLC cells to suppress the proliferation, cell cycle, and glycolytic metabolism and promote the apoptosis of NSCLC cells. Furthermore, rescue experiments revealed that circ-MEMO1 exerted an oncogenic role in NSCLC through targeting miR-101-3p.

KRAS was predicted as a possible target gene of miR-101-3p by StarBase software, and the interaction between KRAS and miR-101-3p was confirmed by dual-luciferase reporter assay. KRAS was a pivotal regulator in the glycolysis of cancer cells (Hu et al., 2012; Ying et al., 2012; Kerr et al., 2016). Besides, KRAS has been identified as an underlying treatment target for cancers (Wu et al., 2019). Chen et al. (2018) found that miR-19a accumulation suppressed the angiogenesis of colorectal cancer through down-regulating KRAS. MiR-216b hampered the progression of pancreatic cancer through suppressing KRAS (Wu et al., 2018). Here, through co-transfecting si-circ-MEMO1 and anti-miR-101-3p into NSCLC cells, we concluded that circ-MEMO1 could up-regulate the expression of KRAS through targeting and down-regulating miR-101-3p in NSCLC cells.

The results of animal experiment suggested that circ-MEMO1 silencing restrained the tumor growth of NSCLC *in vivo*. More *in vivo* studies about the working mechanism of circ-MEMO1 on the progression of NSCLC should be conducted in the future.

Apart from the results that circ-MEMO1 was up-regulated in NSCLC tissues compared with adjacent normal tissues, circ-MEMO1 was also found to be up-regulated in serum-derived exosomes in NSCLC patients compared with healthy volunteers. Serum exosomal circ-MEMO1 might be a promising biomarker for the early diagnosis of NSCLC.

## Conclusion

Our study demonstrated that circ-MEMO1 promoted the progression and glycolysis of NSCLC through up-regulating KRAS via targeting miR-101-3p. Circ-MEMO1/miR-101-3p/KRAS axis might provide a promising target for NSCLC treatment. In addition, exosomal circ-MEMO1 level in serum might be an early diagnostic marker for NSCLC.

## Data Availability Statement

The original contributions presented in the study are included in the article/Supplementary Material, further inquiries can be directed to the corresponding author/s.

## Ethics Statement

The studies involving human participants were reviewed and approved by the protocol in this clinical experiment was authorized by the Clinical Research Ethics Committee of the Henan Provincial Chest Hospital. The patients/participants provided their written informed consent to participate in this study. The animal study was reviewed and approved by animal manipulates were authorized by the Animal Research Ethics Committee of Henan Provincial Chest Hospital.

## Author Contributions

GW and JW designed and supervised the study. CD and GXi conducted the experiments and drafted the manuscript. BZ and HW conducted the experiments and supervised the study. GJ and DC collected and analyzed the data. JS contributed to the methodology and analyzed the data. GXu operated the software and edited the manuscript. All authors read and approved the final manuscript.

## Conflict of Interest

The authors declare that the research was conducted in the absence of any commercial or financial relationships that could be construed as a potential conflict of interest.

## Supplementary Material

FIGURE S1 Circ-MEMO1 overexpression accelerates the proliferation, cell cycle progression, and glycolytic metabolism and suppresses cell apoptosis in H1650 cells. H1650 cells were transfected with vector or circ-MEMO1. **(A)** The overexpression efficiency of circ-MEMO1 was assessed in H1650 cells. qRT-PCR was implemented for the expression detection of circ-MEMO1 in H1650 cells transfected with vector or circ-MEMO1. **(B)** Cell proliferation curve was generated through estimating cell number after transfection for 24, 48, or 72 h via MTT assay. **(C)** Cell cycle progression was analyzed via flow cytometry. The percentages of H1650 cells in G0/G1, S, or G2/M were measured. **(D)** The apoptosis rate of H1650 cells was analyzed using flow cytometry. **(E,F)** The glucose consumption and the production of lactate in circ-MEMO1-overexpressed H1650 cells were analyzed using Glucose Assay Kit and Lactate Assay Kit. **(G)** Western blot assay was applied for protein expression detection of HK2 and LDHA in transfected H1650 cells. ^∗^*P* < 0.05.
