# Supplementary figures and images for: Exosomal Circ-MEMO1 Promotes the Progression and Aerobic Glycolysis of Non-small Cell Lung Cancer Through Targeting MiR-101-3p/KRAS Axis
Source: Front Genet. 2020 Aug 28;11:962. doi: 10.3389/fgene.2020.00962 (PMC7483554; doi:10.3389/fgene.2020.00962)

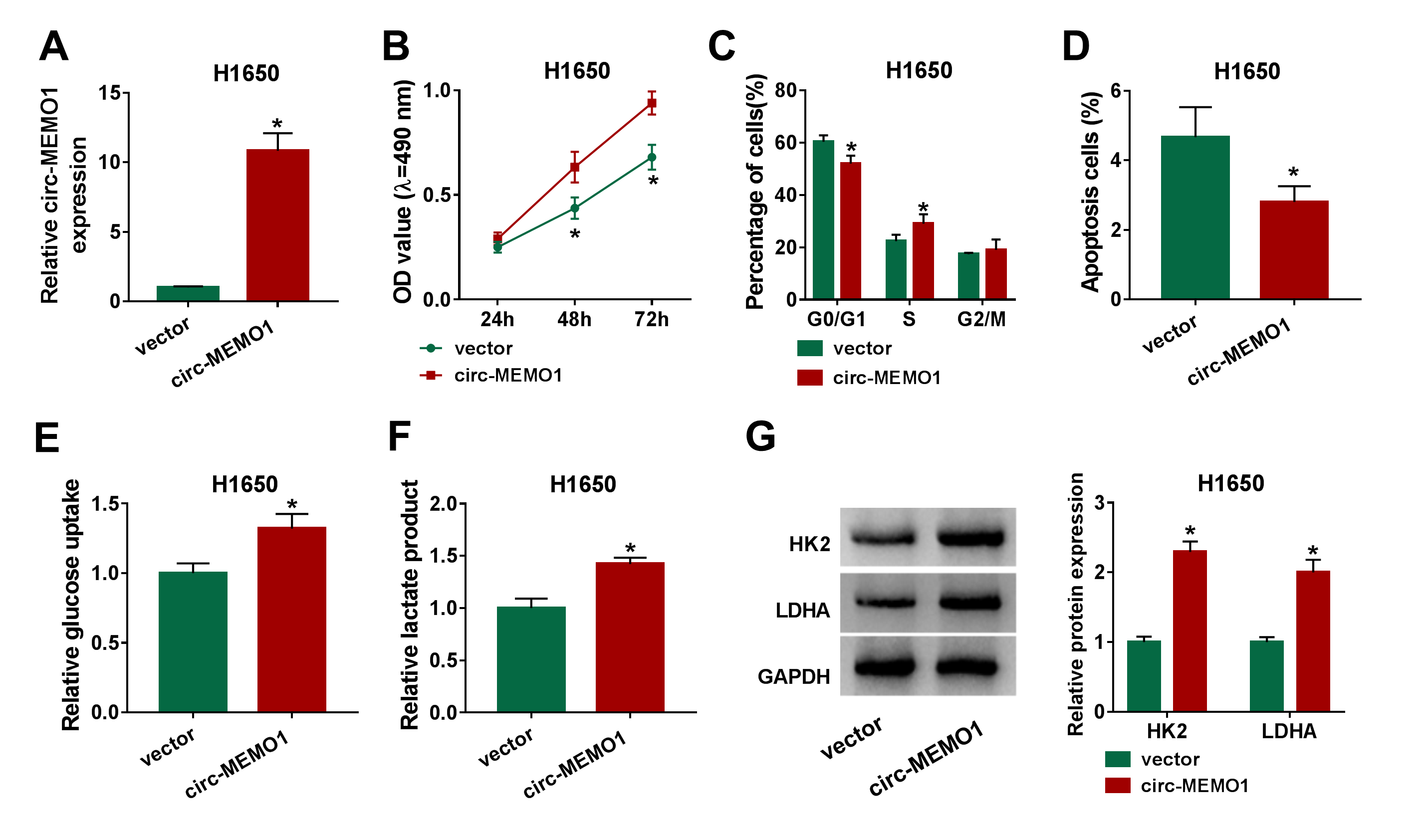

Supplement: FIGURE S1 — Circ-MEMO1 overexpression accelerates the proliferation, cell cycle progression, and glycolytic metabolism and suppresses cell apoptosis in H1650 cells. H1650 cells were transfected with vector or circ-MEMO1. (A) The overexpression efficiency of circ-MEMO1 was assessed in H1650 cells. qRT-PCR was implemented for the expression detection of circ-MEMO1 in H1650 cells transfected with vector or circ-MEMO1. (B) Cell proliferation curve was generated through estimating cell number after transfection for 24, 48, or 72 h via MTT assay. (C) Cell cycle progression was analyzed via flow cytometry. The percentages of H1650 cells in G0/G1, S, or G2/M were measured. (D) The apoptosis rate of H1650 cells was analyzed using flow cytometry. (E,F) The glucose consumption and the production of lactate in circ-MEMO1-overexpressed H1650 cells were analyzed using Glucose Assay Kit and Lactate Assay Kit. (G) Western blot assay was applied for protein expression detection of HK2 and LDHA in transfected H1650 cells. ∗P < 0.05. [file Image_1.TIF]
